# Neuroprotection from protein misfolding in cerebral hypoperfusion concurrent with metabolic syndrome. A translational perspective

**DOI:** 10.3389/fnins.2023.1215041

**Published:** 2023-08-15

**Authors:** Sofía Bordet, Juan Pablo Luaces, Maria Ines Herrera, Liliana Mirta Gonzalez, Tamara Kobiec, Santiago Perez-Lloret, Matilde Otero-Losada, Francisco Capani

**Affiliations:** ^1^Centro de Altos Estudios en Ciencias Humanas y de la Salud, Universidad Abierta Interamericana, Consejo Nacional de Investigaciones Científicas y Técnicas, CAECIHS, UAI-CONICET, Buenos Aires, Argentina; ^2^Centro de Investigaciones en Psicología y Psicopedagogía (CIPP), Facultad de Psicología y Psicopedagogía, Pontificia Universidad Católica Argentina (UCA), Buenos Aires, Argentina; ^3^Departamento de Fisiología, Facultad de Medicina, Universidad de Buenos Aires (UBA), Buenos Aires, Argentina; ^4^Observatorio de Salud Pública, Pontificia Universidad Católica Argentina, Consejo Nacional de Investigaciones Científicas y Técnicas, Buenos Aires, Argentina; ^5^Instituto de Ciencias Biomédicas, Facultad de Ciencias de la Salud, Universidad Autónoma de Chile, Santiago, Chile

**Keywords:** metabolic syndrome, type 2 diabetes, chronic cerebral hypoperfusion, protein misfolding, neurodegeneration, animal models

## Abstract

Based on clinical and experimental evidence, metabolic syndrome (MetS) and type 2 diabetes (T2D) are considered risk factors for chronic cerebral hypoperfusion (CCH) and neurodegeneration. Scientific evidence suggests that protein misfolding is a potential mechanism that explains how CCH can lead to either Alzheimer’s disease (AD) or vascular cognitive impairment and dementia (VCID). Over the last decade, there has been a significant increase in the number of experimental studies regarding this issue. Using several animal paradigms and different markers of CCH, scientists have discussed the extent to which MetSor T2D causes a decrease in cerebral blood flow (CBF). In addition, different models of CCH have explored how long-term reductions in oxygen and energy supply can trigger AD or VCID *via* protein misfolding and aggregation. Research that combines two or three animal models could broaden knowledge of the links between these pathological conditions. Recent experimental studies suggest novel neuroprotective properties of protein-remodeling factors. In this review, we present a summarized updated revision of preclinical findings, discussing clinical implications and proposing new experimental approaches from a translational perspective. We are confident that research studies, both clinical and experimental, may find new diagnostic and therapeutic tools to prevent neurodegeneration associated with MetS, diabetes, and any other chronic non-communicable disease (NCD) associated with diet and lifestyle risk factors.

## 1. Introduction

Metabolic syndrome (MetS) is a cluster of cardio-cerebrovascular risk factors including at least three of the following: abdominal obesity, hyperglycemia, hypertriglyceridemia, hypertension, and insulin resistance (American Diabetes Association, 2002; Bruce and Hanson, 2010; McCrimmon et al., 2012; Otero-Losada et al., 2016a). Based on clinical and experimental evidence, metabolic syndrome (MetS) and type 2 diabetes (T2D) are considered risk factors for chronic cerebral hypoperfusion (CCH) and neurodegeneration. The particularly risky conditions in MetS are due to the coexistence of atherogenic dyslipidemia (high triglycerides and LDL with low HDL cholesterol blood concentrations), hypertension, hyperglycemia, a prothrombotic state, and a proinflammatory state (Grundy, 2008; Russo, 2012; Barale and Russo, 2020; Kazamel et al., 2021). Typical of Mets is chronic subclinical vascular inflammation in which abnormal production of adipocytokines such as tumor necrosis factor α, interleukin-1 (IL-1), IL-6, leptin, and adiponectin develops, eventually leading to atherosclerotic processes (Farooqui et al., 2011; Kwon and Pessin, 2013; Opatrilova et al., 2018; Kim et al., 2022).

Silently, MetS and T2D lead to microvascular dysfunction and CCH. Laboratory-anthropometric measurements have supported the interplay between MetS, T2D, and CCH, as well as their association with Alzheimer’s Disease (AD) and/or Vascular Cognitive Impairment and Dementia (VCID). Similarly, preclinical assessments have contributed to the knowledge of the etiology of VCID and AD, suggesting protein misfolding and aggregation as common pathophysiological features (Neuropathology Group of the Medical Research Council Cognitive Function and Ageing Study, 2001; Duncombe et al., 2017).

Different animal paradigms have been developed to reproduce the MetS as it occurs in humans. Recently, the number of experimental studies resembling MetS, T2D, and CCH has increased significantly (Herrera et al., 2020). Translational investigations have explored the extent to which MetS or T2D can cause CCH, using different markers of cerebral blood flow (CBF). In addition, recent studies have attempted to unravel the different mechanisms of neurodegeneration by combining two or three murine models. In recent years, research has also contributed to the discovery of potential neuroprotective strategies targeting the proteostasis network (Yumei et al., 2017; Zhang et al., 2020; Zheng et al., 2021), i.e., the machinery for biogenesis, folding, conformational maintenance and degradation of proteins with molecular chaperones as key coordinators. In this context, the present work will update the scientific knowledge following the single and combined experimental induction of MetS, T2D, and CCH, after analyzing the different rodent models of these chronic diseases. This review revisits and summarizes the neuroprotective properties of protein-remodeling factors based on new evidence. This translational update will help scientists and health professionals to redirect therapeutic and preventive strategies not only to include but especially to emphasize, the correction of unhealthy lifestyle habits, the avoidance of brain derangement, and the deterioration of mental health.

## 2. MetS, T2D, and CCH models. A translational overview

### 2.1. MetS

The most representative rat strain for the study of MetS is the Obese Zucker rats, in which animals show similar changes to those observed in humans (de Artiñano and Castro, 2009). In 1961, Lois Zucker developed this rat strain by mutating leptin, which leads to obesity from the third week of life (Phillips et al., 1996). The upregulation of this hormone, which is synthesized in adipose tissue and acts on brain receptors (Ahima and Flier, 2000), forms the molecular mechanism of the characteristic phenotype of the Zucker rat strain: hyperphagia, deposition of energy in adipose tissue, dyslipidemia, mild glucose intolerance, hyperinsulinemia, and vascular changes (Picó et al., 2002; de Artiñano and Castro, 2009). Unlike Obese Zucker rats, the Wistar Ottawa Karlsburg W (WOKW) model does not cause cardiovascular disease by a single gene mutation. It thus resembles pathological conditions in the clinical context. The disadvantage of this preclinical approach is that it reproduces MetS between 8 and 10 weeks of life (de Artiñano and Castro, 2009).

Other experimental methods aim to profile anomalies in patients with MetS. Sweet, carbonated beverage, high-fat diet (HFD), and low-capacity runner (LCR) models focus on environmental variables in the etiology of MetS, specifically dietary and lifestyle risk factors (Abdel-Megeid et al., 2011). The latter is based on the development of cardiovascular disease after the artificial induction of low aerobic capacity (Wisløff et al., 2005). LCR rats are selected according to their efficiency in a running task and represent the group that can run short distances due to low intrinsic aerobic ability. These animals are bred together until they reach the 11th generation, where elevated blood pressure, insulin resistance, hyperinsulinemia, and endothelial dysfunction are observed (de Artiñano and Castro, 2009). HFD rats have impaired glucose tolerance (IGT) and T2D, as they consume approximately 58% of the total energy supply from increased energy intake, in addition to lower metabolic efficiency during the first week of life (Winzell and Ahrén, 2004). In the sweet, carbonated beverage model, 6 months of *ad libitum* consumption of this liquid source leads to hyperglycemia, hypertriglyceridemia, hypercholesterolemia, overweight, systolic hypertension, cardiac and renal changes, and oxidative stress (Otero-Losada et al., 2011, 2013, 2014, 2015, 2016a,b).

Finally, alternative models of MetS are derived from the model of choice for hypertension: the spontaneously hypertensive rat (SHR). Indeed, corpulent SHR rats exhibit hypertriglyceridemia and abdominal obesity. In particular, the obese SHR, also known as the Koletsky rat after its creator, shows early vascular pathology. In this way, it resembles human atherosclerosis (Kastin et al., 1999). Instead, the SHR/N corpulent model was developed to mimic obesity and non-insulin-dependent diabetes mellitus (NIDDM). SHR/NDmc-corpulent rats are characterized by obesity, together with hyperphagia and metabolic alterations. And stroke-prone-SHR fatty rats present high levels of hypertension, which provokes atherosclerosis and stroke (Michaelis et al., 1989).

### 2.2. Rodent models of diabetes type 2

Obesity and diabetes are known risk factors for the development of dementia, including different neurodegenerative diseases (Livingston G. et al., 2020; Livingston J. M. et al., 2020). Mimicking human obesity and its co-morbidities in animal models is a complex task. The most widely used models are murine and reproduce at least some features of obesity (Lutz, 2020). Type 2 diabetes mellitus (T2D) is one of the most damaging consequences of obesity. It is a multifactorial disorder, but obesity-induced insulin resistance accelerates the onset of diabetes (Kleinert et al., 2018). Individuals diagnosed with T2D and dementia present vascular pathology, small stroke, and Alzheimer’s disease (AD)-related neuropathologies (Gooch and Wilcock, 2016). Animal models of T2D usually consist of genetic manipulation, exposure to obesogenic diets, or other manipulations such as chemical administration (King and Bowe, 2016; Lutz, 2020).

Leptin-deficient OB/OB mice and leptin receptor-deficient db/db mice are two monogenic rodent models that have been widely studied and used as preclinical models that mimic the conditions of obesity and T2D development (Coleman, 1978; Kleinert et al., 2018; Suriano et al., 2021). The leptin receptor is key to these models due to its role in the regulation of body weight and food intake (Ramos-Lobo and Donato, 2017). In both models, the leptin receptor pathway is affected, leading to hyperphagia, massive obesity, and fat mass gain. However, they show significant differences. For example, there are several differences in the inflammatory tone in different tissues. The OB/OB breed showed altered hepatic metabolism, and therefore hepatic dysfunction, in contrast to db/db mice, which have a higher inflammatory tone in adipose tissue (Suriano et al., 2021).

To create a model of Vascular Cognitive Impairment and Dementia (VCID), Niedowicz et al. (2014) crossed an obese db/db mouse with a knock-in AD model. It showed morbid obesity and glucose intolerance from an early age and developed cognitive impairment at 12 months of age. In addition, this model displayed beta-amyloid plaques and vascular pathologies in the absence of HFD (Niedowicz et al., 2014). Another TDM and dementia model was obtained by crossing an APP23 AD mouse with a diabetic OB/OB strain. It showed learning deficits and histopathological changes, such as vascular inflammation, at 8 weeks of age (Takeda et al., 2010; Gooch and Wilcock, 2016).

Another monogenic model, independent of the leptin pathway, is the Otsuka Long-Evans Tokushima Fatty Rats (OLETF) (Lutz, 2020). Their clinical and pathological features are late-onset hyperglycemia, mild obesity, male inheritance, renal complications, and a chronic course of the disease (Kawano et al., 1992).

Induced T2D can be achieved chemically by β-cells ablation in murine models using diabetogenic chemicals, such as streptozotocin (SZT; Szkudelski, 2012; Pandey and Dvorakova, 2020). STZ is one of the most widely used diabetic models in the literature over the last decade (Pandey and Dvorakova, 2020; Furman, 2021). This model mimics many typical signs and symptoms of human diabetes such as increased food and water intake, excessive weight gain, elevated blood glucose concentrations, diastolic cardiac dysfunction, cataracts, and neuropathy (Wei et al., 2003). STZ also affects the peripheral and central nervous system, reproducing an insulin-resistant brain condition (Goyal et al., 2016). As a result, the animal develops cognitive and cholinergic deficits, glucose hypometabolism, oxidative stress and neurodegeneration similar to sporadic Alzheimer’s disease in humans (Haluzík and Nedvídková, 2000).

Diet-induced obesity, which includes HFD models, is widely used to study the interaction between diet and genes in obesity and insulin resistance (Obrosova et al., 2007; Kleinert et al., 2018). However, as with monogenic models, there are some limitations in understanding and treating human obesity and diabetes. For example, studies using HFD models show little similarity to human pathophysiology and differ in methodological aspects such as diet composition, rodent strain, and gender, and (Lai et al., 2014; Kleinert et al., 2018). On the other hand, although monogenic models have proven useful for pharmacological and genetic studies, they lack environmental factors. As a result, the pathogenesis is far from clinical diabetes (Karasawa et al., 2009).

HFD/STZ is a murine model that aims to combine their features to approximate clinical situations in humans (Zhang et al., 2008). HFD causes hyperinsulinemia and insulin resistance, while STZ administration leads to a severe reduction in functional beta cells, culminating in pancreatic dysfunction (Skovsø, 2014). In the brain, HFD/STZ rats showed ischemia, followed by reduced cognitive functions due to higher levels of β-amyloid caspase-3 positive cells in the hippocampus (Zhang et al., 2009).

### 2.3. CCH

A sudden interruption of CBF is associated with stroke, whereas a progressive and silent decrease in CBF is produced in CCH. Blood hypoperfusion is a precursor of neurodegenerative diseases such as AD. Several preclinical models of CCH have been developed to study reductions in CBF. Some of them resemble focal ischemia, which represents the pathophysiology of stroke. In these models, a specific vessel is occluded and CBF is reduced by approximately 70%. In general, transient or permanent occlusion of the middle cerebral artery is used. Multiple infarcts can be mimicked either by intra-arterial injection of emboli or by spontaneous stroke induction (SHRSP). Global ischemia paradigms reduce CBF to a greater extent, including transient common carotid artery occlusion (TCAO), three-and four- vessel occlusion, and cardiac arrest (Duncombe et al., 2017).

If researchers intend to reduce CBF to a lesser extent (by 30–50%) to study CCH, ligation or occlusion of the unilateral or bilateral common carotid arteries (two-vessel occlusion) can be performed. The reproduction of a subtle but persistent CBF drop becomes relevant to the study of VCID. Bilateral common carotid artery occlusion (BCCAO) can be refined to mimic modest reductions in CBF. Bilateral common carotid artery stenosis (BCCAS) has been established to reduce flow to 50% of baseline (Shibata et al., 2004). In addition, aneroid devices can be used to mimic progressive stenosis without CBF recovery. In this way, extracellular fluid is absorbed, leading to arterial constriction and a gradual onset of hypoperfusion. This model is known as gradual common carotid artery stenosis (Kitamura et al., 2012). Therefore, rodent paradigms of cerebral hypoperfusion vary from traditional acute occlusion protocols to gradual and slower stenosis methods (Duncombe et al., 2017). These gradual mechanisms are the ones that better mimic the clinical profile of CCH. However, experimental stenosis involves challenging techniques, with BCCAO being the model of choice (Zhao and Gong, 2015). An alternative approach implies asymmetric common carotid artery surgery.

Progressive occlusion of the right carotid artery lasts 1 month, while the left artery is subjected to 50% stenosis by placing a micro-coil. Further research may help to develop CCH on a larger scale (Duncombe et al., 2017).

### 2.4. Do Mets or T2D lead to CCH? Discussing evidence within different animal models

The characterization of some pathological mechanisms by which MetS or T2D leads to a decrease in cerebral blood flow was performed using murine animal models, and CCH was assessed using various markers. Reduced perfusion of the cerebral circulation acts as a mechanism that increases the risk of dementia in the context of metabolic dysregulation.

Clinical studies have shown a reduction in cerebral perfusion in patients with hyperglycemia, through the presence of microvascular changes (van Golen et al., 2013). Preclinical models are needed to understand pathological mechanisms. Animal models can be used to assess different experimental designs, with the possibility of trying specific diets and exposure times (Livingston G. et al., 2020; Livingston J. M. et al., 2020). In addition, more studies are needed to explain moderate and chronic forms of cerebral vascular impairment, such as metabolic diseases, which are necessary to characterize how cognitive decline may develop by accelerating neurodegeneration.

The Zucker obese (ZO) rat model has been used to study the cerebrovascular consequences of insulin resistance, showing that the mitochondrial function contributes to impaired vasodilation in ZO rats (Katakam et al., 2009). Other authors using the OZR model demonstrate a lumen reduction in the middle cerebral artery lumen in OZR rats compared to controls (Osmond et al., 2009).

Using the db/db diabetic mice model (the leptin receptor gene mutant model) combined with the CCAO model, brain perfusion was examined using doppler optical coherence tomography and angiography. The vascular condition was impaired in db/db mice, which showed a perfusion deficit compared to control animals. These results indicate that T2DM increases cerebral hypoperfusion in the diabetic mice model (Nishijima et al., 2016).

Another model of MetS was tested using a cafeteria diet in Sprague Dawley rats and cerebral perfusion in the hippocampus was measured using dynamic contrast-enhanced magnetic resonance imaging (MRI). After three to four months of treatment, the ultra-processed diet produced a MetS phenotype and increased resting cerebral perfusion in the cerebral cortex and hippocampus but decreased cerebrovascular reactivity (Gomez-Smith et al., 2018). In another experiment, the cafeteria diet was administered for 30 weeks, but no dietary effects on the forebrain and hippocampal blood flow were observed after the experimental period (Livingston G. et al., 2020; Livingston J. M. et al., 2020).

Knowledge of how obesity leads to an increase in CCH was obtained from models of HFD-based obesity in mice. The authors showed the effect of HFD, which produces endothelial dysfunction of cerebral arterioles after 12 and 36 weeks of treatment (Lynch et al., 2013). In other mice HFD models, no differences were observed after 6.5 months of treatment when cerebral blood flow was measured by MRI. However, HFD treatment impaired blood flow in UCCAO mice compared to the low-fat diet group (Zuloaga et al., 2016).

## 3. Updated findings on neuroprotective properties of protein-remodeling factors

Rodent models of CCH have been used to investigate how prolonged oxygen deprivation can promote protein aggregation in AD or VCID (Herrera et al., 2020). Unlike stroke models with sudden interruption of blood supply, CCH is achieved by a gradual decrease in cerebral blood flow. Little is known about how CCH affects protein homeostasis. We summarize work analyzing proteostasis markers in rodent models of CCH.

Models of vascular cognitive impairment based on CCH showed loss of neuronal function with different alterations along the protein synthesis process (Herrera et al., 2020).

Three different mechanisms activate proteolytic pathways to eliminate misfolded proteins: (1) Ubiquitin (Ub)-proteasome system (UPS), consisting of a proteasome that removes proteins conjugated to Ub; (2) Chaperone-mediated autophagy (CMA), in which substrates recognized by the chaperone heat shock cognate 70 (Hsc70) are eliminated by lysosomal enzymes; (3) Macroautophagy, in which proteins that evade surveillance form aggregates, are incorporated into autophagosomes and eliminated by lysosomal activity (Ciechanover and Kwon, 2015).

In recent years, there have been relevant findings on protein misfolding as a target of CCH aiming at clearance mechanisms (Herrera et al., 2018a,b). Autophagy is a degradation system of damaged proteins that acts through autophagosomes, double-membrane vesicles that capture cytosolic cargo and deliver it to the lysosome (Choi et al., 2013; Ciechanover and Kwon, 2015). It is a cellular process that has the potential to be deleterious and may play a role in CCH (Herrera et al., 2018a). Gastrodin (GAS), a bioactive component of the herb TianMa, ameliorated learning and memory impairment in a rat model of vascular dementia. GAS promoted autophagy flux *via* inhibition of the Ca2+/CaMKII signaling pathway (Chen et al., 2021). Hippocampal degeneration was ameliorated by the administration of Lipoxin A4 methyl ester (LXA4 ME) in CCH caused by BCCAL and 2VO in rats. This effect has been attributed to the regulation of endoplasmic reticulum stress and macroautophagy (Jia et al., 2015). Baclofen has also shown protective potential against the effects of CCH. Chronic treatment with this drug prevented hippocampal atrophy and neuronal apoptosis by suppressing cytodestructive autophagy activity *via* the protein kinase B (Akt)/ERK- B-cell lymphoma 2 (Bcl2)-beclin-1 signaling pathway and positive regulation of protective autophagy, which activates the ionotropic metabotropic γ-aminobutyric acid (GABA)A receptor-connexin (CX)43/CX36 signaling pathway (Liu et al., 2015). Another example of neuroprotection through modulation of autophagy is treatment with the fatty acid amide hydrolase (FAAH) URB597 inhibitor. In a mouse model of CCH by BCCAO, treated animals showed suppressed apoptosis and amelioration of ultrastructural neurodegeneration and cognitive impairment in CA1 *via* the m-TOR pathway (Wang et al., 2017).

The WFS1 gene is known to cause Wolfram syndrome, a neurodegenerative disease characterized by diabetes insipidus, diabetes mellitus, optic atrophy, deafness, and other neurological and psychiatric problems (Chen et al., 2023). Ubiquitin-mediated proteasomal degradation is activated after a deleterious post-translational modification (Li et al., 2020). Brain WFS1 is highly expressed in the amygdala, hippocampus, olfactory tubercles, brainstem nuclei, and thalamic reticular nucleus (Li et al., 2020). Wolframin, the protein encoded by this gene, is mainly involved in membrane trafficking, in the endoplasmic reticulum (ER) Ca2+ homeostasis (Gong et al., 2021; Chapla et al., 2022) and the regulation of unfolded protein response (UPR) in various cell types (Li et al., 2020). In particular, WFS1 deficiency has been reported to be involved in the upregulation of three major UPR pathways initiated by ER membrane sensors: protein kinase RNA-like endoplasmic reticulum kinase (PERK), inositol-requiring enzyme 1 (IRE1), and the activating transcription factor 6 (ATF6) (Hetz and Smita, 2017; Da Silva et al., 2020; Fu et al., 2023). Different mutations in WFS1 have been associated with high production of proinflammatory cytokines by peripheral blood mononuclear cells, particularly TNF-α, IL-1β, and IL-6 (Panfili et al., 2021). Tau protein aggregates have been found in WFS1-expressing excitatory neurons in the entorhinal cortex, one of the earliest affected regions in AD. In individuals with early stages of Braak-associated AD, there is an enrichment of genes related to chronic ER stress and the autophagy-lysosome pathway (ALP) in excitatory neurons expressing high levels of WFS1 (Li et al., 2020). These levels have an impact on disease manifestation,showing that deficiency is associated with increased tau pathology and neurodegeneration. It has also been reported that overexpression of WFS1 can reduce these changes (Chen et al., 2022). WFS1 is being considered a new therapeutic target for different neurodegenerative diseases, particularly Alzheimer’s disease (Chen et al., 2023).

The ubiquitin-proteasome system (UPS) is an ATP-consuming proteolytic system that is another protein misfolding clearance mechanism. The proteasome causes selective degradation by the conjugation of ubiquitin (ub). Ub ligases and chaperones recognize folding abnormalities, beginning the degradation process (Ciechanover and Kwon, 2015). In a murine model of BCCAO, animals were treated with melatonin. As a result, CCH-induced stress protein expression was modulated and levels of the chaperone HSP70 in the hippocampus were restored (Ozacmak et al., 2009). Endogenous endocannabinoid anandamide (AEA) analogs have potential neuroprotective properties by regulating the UPS (Hai et al., 2013; Herrera et al., 2018a,b). In a rat model, N-stearoyl-L-tyrosine (NSTyr) increased proteasome peptidase activity, inhibited intracellular aggregation of ubiquitinated proteins, and thereby protected the animals’ hippocampus as a result (Hai et al., 2013). Palmitoylethanolamide, another AEA analog, reduced alterations in hippocampal MAP-2 levels and reversed behavioral dysfunctions in a murine acute model of hypoxia (Herrera et al., 2018a,b).

## 4. Concluding remarks and future perspectives for the prevention of non-communicable brain diseases

The association between MetS and CCH may lead to vascular cognitive impairment. However, the characterization of the MetS metabolic changes leading to cognitive impairment remains to be understood. Promising new research into agents that promote proteome homeostasis may ameliorate vascular cognitive dysfunction. Different models of CCH resulted in cognitive impairment and alterations in protein synthesis. Misfolded oligomers are responsible for the cytotoxicity that leads to neuronal death from protein aggregates in many neurodegenerative diseases. Substantial clinical benefits may be achieved by increasing the knowledge of the cellular pathways involved in the degradation of pathogenic proteins. Studies on the development of proteostatic changes in the context of MetS and CCH using animal models will shed light on the mechanisms involved in the establishment of VCID and AD.

Protein-remodeling factors should be assessed as neuroprotective agents for the prevention of cerebrovascular disease and cognitive decline. Although lifestyle and dietary changes are the main approaches to improving the MetS condition, neuroprotective agents can be used simultaneously to prevent the disruption of protein synthesis and cell death.

## Author contributions

JL, MO-L, SP-L, SB, TK, and MH: writing – original draft. JL, SB, and MH: writing – review and editing. SB, JL, MG, MO-L, and MH: conceptualization. FC: funding acquisition. FC, MO-L, and SP-L: supervision. All authors contributed to the article and approved the submitted version.

## Funding

This work was supported by grants to FC from COFECyT PF103 2022–262,025, PS1 UAI 2023–2025, UK 2023–2025, PICT-2020-SERIEA-01926.

## Conflict of interest

The authors declare that the research was conducted in the absence of any commercial or financial relationships that could be construed as a potential conflict of interest.

## Publisher’s note

All claims expressed in this article are solely those of the authors and do not necessarily represent those of their affiliated organizations, or those of the publisher, the editors and the reviewers. Any product that may be evaluated in this article, or claim that may be made by its manufacturer, is not guaranteed or endorsed by the publisher.
